# Establishing Evidence Criteria for Implementation Strategies: A Delphi Study for HIV Services

**DOI:** 10.21203/rs.3.rs-3979631/v1

**Published:** 2024-02-29

**Authors:** Virginia Mckay, alithia zamantakis, Ana Michaela Pachicano, James Merle, Morgan Purrier, McKenzie Swan, Dennis Li, Brian Mustanski, Justin D Smith, Lisa Hirschhorn, Nanette Benbow

**Affiliations:** Washington University in Saint Louis; Northwestern University; Northwestern University; The University of Utah; Northwestern University; Washington University in St Louis; Northwestern University; Northwestern University; The University of Utah; Northwestern University; Northwestern University

**Keywords:** HIV, evidence-based intervention, implementation science

## Abstract

**Background.:**

There are no criteria specifically for evaluating the quality of implementation research and recommend implementation strategies likely to have impact to practitioners. We describe the development and application of the Best Practices Rubric, a set of criteria to evaluate the evidence supporting implementation strategies, in the context of HIV.

**Methods.:**

We developed the Best Practices Rubric from 2022–2023 in three phases. (1) We purposively selected and recruited by email participants representing a mix of expertise in HIV service delivery, quality improvement, and implementation science. We developed a draft rubric and criteria based on a literature review and key informant interviews. (2) The rubric was then informed and revised through two e-Delphi rounds using a survey delivered online through Qualtrics. The first and second round Delphi surveys consisted of 71 and 52 open and close-ended questions, respectively, asking participants to evaluate, confirm, and make suggestions on different aspects of the rubric. After each survey round, data were analyzed and synthesized as appropriate, and the rubric and criteria were revised. (3) We then applied the rubric to a set of research studies assessing 18 implementation strategies designed to promote the adoption and uptake of pre-exposure prophylaxis, an HIV prevention medication, to assess reliable application of the rubric and criteria.

**Results.:**

Our initial literature review yielded existing rubrics and criteria for evaluating intervention-level evidence. For a strategy-level rubric, additions emerged from interviews, for example, a need to consider the context and specification of strategies. Revisions were made after both Delphi rounds resulting in the confirmation of five evaluation domains – research design, implementation outcomes, limitations and rigor, strategy specification, and equity – and four evidence levels – best practice, promising practice, more evidence needed, and harmful practices. For most domains, criteria were specified at each evidence level. After an initial pilot round to develop an application process and provide training, we achieved 98% reliability when applying the criteria to 18 implementation strategies.

**Conclusions.:**

We developed a rubric to evaluate the evidence supporting implementation strategies for HIV services. Although the rubric is specific to HIV, this tool is adaptable for evaluating strategies in other health areas.

## Introduction

Implementation science is dedicated to improving the uptake and use of evidence-based interventions, practices, and policies to capitalize on scientific knowledge and impact human health. Central to the goals of implementation research is building the evidence for implementation strategies, defined as techniques or change efforts to promote the adoption, implementation, and sustainment of evidence-based interventions (EBIs) (1). In a recent review, scholars within the field of implementation science recognized that a more robust research agenda related to implementation strategies is needed to yield the promised benefits of improved EBI implementation for practitioners (2). Within this agenda was a call for more research on the effectiveness of implementation strategies. Expanding on this priority, criteria on which to evaluate evidence quality are needed to assess whether the evidence supporting the effectiveness of any given strategy is sufficient. Without criteria on which to evaluate implementation research focusing on strategies, it is difficult to recommend strategies that are likely to be the most valuable for practitioners or to identify strategies that may hold initial promise but would benefit from more robust research. Criteria are also an essential element of the creation of a compendium of evidence-based implementation strategies, which is a key dissemination approach for delivering evidence to implementers.

At the intervention level, criteria and rubrics are available to synthesize research outcomes and evaluate research quality behind the evidence supporting an intervention and make recommendations about their use, such as Grading of Recommendations Assessment, Development, and Evaluation (GRADE) or that used by the United States Preventative Services Task Force (3, 4). These guidelines often consider different domains of research outcomes and quality, like the health outcomes, the research design, and potential for bias in the outcomes because of the research design. Based on these guides, health institutions, like the Preventative Services Task Force, make recommendations about the best interventions across a wide set of health conditions to assist providers and organizations in making clinical and policy-level decisions. To our knowledge, no equivalent set of criteria for implementation strategies are available. As such, it is difficult to discern the quality of evidence supporting an implementation strategy and whether strategies should be recommended to practitioners to support the implementation of EBIs.

Existing criteria, like GRADE, may serve as a valuable starting point for building criteria applicable to the field of implementation research (5). Effectiveness research and associated evaluation criteria, which heavily emphasizes internal validity, considers the highest quality evidence to be from research designs like double-blind randomized control trials. In implementation research, internal validity tends to be more balanced with external validity so that the results are generalizable to target communities. With external validity in mind, implementation research is typically conducted in practice settings and involves assessment of the organizations and providers who will be impacted by the implementation strategy and subsequently the intervention under consideration. As a result, it is often inappropriate, impractical, and/or undesirable to leverage research designs like randomized controlled trials, because it is not possible to blind practitioners to the strategy and/or intervention or randomize at the unit of analysis (6–8). These realities make direct application of intervention-level criteria inappropriate - necessitating criteria specific to the field (3).

### HIV and Implementation Research

We describe our efforts to develop a set of criteria and evaluation process for implementation strategies to address the HIV epidemic in the United States. Improvements in the US HIV epidemic have been modest over the last two decades, with disparities among communities disproportionally affected by HIV increasing (9). In an attempt to address HIV incidence, the Centers for Disease Control and Prevention have curated a repository of EBIs to support HIV prevention since the early 2000s and supported dissemination and implementation of a subset of these (10). Furthermore, major biomedical advancements such as pre-exposure prophylaxis (PrEP), have proven to be very effective at preventing HIV. Yet many of these interventions have not been widely implemented with equity to yield their intended benefit. Only an estimated 30% of individuals who would benefit from PrEP receive it, with growing disparities by race, gender, income, citizenship status, and intersectional marginalization (11–14). Uptake and adherence remain suboptimal along the HIV care continuum (i.e., prevention, testing, diagnosis, linkage-to-care, and treatment), indicating, in part, failed implementation and opportunities to develop evidence-informed implementation strategies (11). In 2019, the Ending the HIV Epidemic (EHE) Initiative was launched as a coordinated effort among several federal agencies to address HIV-related implementation problems. In alignment with EHE, the National Institutes of Health supported a number of mechanisms and projects to conduct research on implementation strategies (15). With the growing mass of HIV-related implementation research has come an equally growing knowledgebase focusing on numerous implementation strategies targeting multiple aspects of the HIV care continuum, in a wide scope of settings, evaluating various implementation outcomes (16).

In an effort to create, synthesize, and disseminate generalizable knowledge, the Implementation Science Coordination Initiative (ISCI) was funded by the National Institutes of Health to provide technical assistance in implementation research funded by the EHE Initiative, coordinate research efforts, synthesize literature through systematic reviews, develop tools to assist researchers, and disseminate synthesized and evaluated research findings to researchers, policymakers, providers, and more (17, 18). As part of this effort, we developed a rubric to evaluate the level of evidence of HIV-related implementation strategies to identify best-practice strategies that can promote effective implementation and uptake of EBIs by HIV practitioners nationwide.

## Methods

### Overview

We conducted the project in three phases: 1) a literature review in tandem with key informant interviews to generate criteria for our tool, 2) a modified Delphi to evaluate and revise our initial tool and criteria; 3) a pilot application of our rubric to a set of implementation research studies. Delphi data were collected from March 2022 to June 2023. Piloting occurred in the fall of 2023. Our data collection protocol was reviewed by the Institutional Review Board at Northwestern University and determined to be non-human subjects research. All data collection instruments have been included as a supplemental file (Supplemental File A), and data are available in a de-identified format from the first author on reasonable request. Methods and results are reported according to STROBE reporting guidelines (Supplemental File B).

### Key Informant Interviews and Literature Review

We first conducted a review of the scientific and grey literature of existing compilations of criteria for assessing EBIs. We utilized this literature to construct an interview guide for key informant experts with questions focusing broadly on informants’ perceptions of the state of the field of HIV implementation research and key points that would need to be considered for or incorporated into a tool to assess evidence to define best practices in implementation strategies. We identified, recruited, and interviewed a range of experts, including implementation scientists, HIV providers and implementers, and representatives from related fields of public health research (e.g., quality improvement), and public health agency officials. All interviews were scheduled in the Spring of 2022 and were approximately 30–45 minutes long. Interviews were recorded and transcribed via Zoom. Two Ph.D.-level researchers with expertise in qualitative and mixed methods research performed an inductive, thematic process of analysis to explore patterns and categorize responses. Based on their responses, we iteratively developed a preliminary tool, criteria, and decision diagram for evaluating the quality of implementation strategy research.

### Modified Delphi

#### Identification and Recruitment of Delphi Participants.

We conducted an asynchronous, modified Delphi with participants of similar expertise as our key informants in two rounds. Participants were recruited using snowball recommendations from those that were interviewed as key informants. Our eligibility criteria included fluent English speakers and those working in the fields of HIV, mental health, substance misuse, social services, primary care, women’s health, or other related areas of public health. If participants were unable to complete the survey, an alternative contact could be recommended. After this first invitation, we sent semiweekly reminder emails for six weeks. A $10 gift card was given to participants for completing the first survey, and a $50 gift card was given to participants for completing the second survey.

#### Data Collection and Measures.

The surveys were implemented using Qualtrics. The surveys were piloted with members of the ISCI research team to ensure question clarity. Each survey took participants approximately 45–75 minutes to complete.

#### First-round Delphi instrument.

This survey consisted of 71 items. The goal was to generate consensus about which aspects of the tool were most important and least important and whether we had included all the elements that participants felt were necessary. The first portion of the survey gathered demographic and basic information about the participant (e.g., age, race, ethnicity, gender), characteristics of the participant’s work (e.g., I work primarily in… select all areas that apply”), as well as the participant’s experience in implementation research (e.g., How would you describe your knowledge level of implementation science?).

The second portion of the survey evaluated proposed domains (Overall Evidence of Effectiveness, Study Design Quality, Implementation Outcomes, Equity Impact, Strategy Specification, and Bundled strategies) and corresponding criteria. Participants were asked to agree or disagree (Yes/No) with the adding/dropping/combining of domains; this was followed by an open-ended question asking why they agreed to said addition/dropping/combining (if applicable). This portion also contained two 5-point Likert-type scales asking participants to rank the domains in order from most important to least important. The third portion of the survey was aimed at gaining the participant’s opinion on the specific criteria (e.g., effect size and effect direction for implementation outcomes) within each domain. For each domain, the participant was asked if there were any criteria that needed to be added/dropped (Yes/No), followed by an open-ended question asking why they would like these items added/dropped (if applicable). The participant was then provided a 5-point Likert scale in which they ranked each item from “Very unimportant” to “Very important”. These questions were repeated for all criteria in all domains.

The final portion of the survey was where the Levels of Evidence (Best Practice Strategy, Promising Strategy, Emerging Strategy, Undetermined Strategies, and Not Recommended Strategy) and their definitions were introduced. The participant was asked if there should be any adding/dropping/combining of the evidence levels (Yes/No), followed by an open-ended question asking why they would like these evidence levels to be added/dropped/combined (if applicable).

#### Second-round Delphi instrument.

This survey consisted of 52 items. As all participants from Round 2 were recruited from Round 1, the goal of this was to test and receive feedback on the changes to the tool made in response to the results of Round 1. The first portion of the survey gathered the same demographic and basic information as in the first round. The second portion consisted of an overview of the updated tool, including definitions of the domains, criteria, and levels of evidence, and asked for feedback on changes made from the Round 1 results. For example, in the first round of the Delphi survey, participants responded that they would like for greater specificity within the criteria of the Study Design domain. As a response, we split this domain into two domains for Round 2: “Study Design” and “Study Rigor and Limitations.” We presented this change to the participant and asked them to agree or disagree with this change (Yes/No); if “No” was selected, this prompted an open-response question asking for further explanation. Lastly, we asked respondents to attempt to apply the criteria and give an evidence-level rating to a set of fictional cases of implementation research studies, and then allowed respondents to comment on the application and rating process.

#### Data Analysis and Management.

Quantitative data were managed and analyzed in Excel. Quantitative data were analyzed descriptively, primarily as percent agreement or disagreement for domains, evidence levels, and individual criteria within domains. Qualitative data were analyzed in Dedoose software and Excel, using a rapid direct qualitative content analysis approach (19). Qualitative data were analyzed by a Ph.D.-level researcher with qualitative research expertise and were intended to confirm or complement quantitative analyses.

### Pilot and Application to PrEP Implementation Strategies

To ensure a high-quality process for reviewing literature and consistent application of criteria, we piloted the tool with a set of implementation strategies designed to promote the uptake of evidence-based HIV services. After two trainings, four Ph.D.-level members of the ISCI team who were also engaged in systematic reviews of HIV literature applied the criteria to an existing set of eight papers reporting on implementation strategies designed to promote PrEP uptake (20) coding a rating for each criteria. We calculated agreement as a simple percentage of identical ratings between two coders out of the total number of criteria, domain ratings, and overall rating (40 items).

## Results

We report the primary results from each stage of our process as well as significant changes to the rubric made at each stage.

Literature Review and Key Informant Interviews Our initial literature review yielded several existing rubrics, tools, criteria and processes for evaluating evidence supporting a specific intervention (5,21). Many had a similar structure of grouping criteria by domain (e.g., aspects of the research design or strength of the outcomes) and having different evidence ratings or levels (e.g., low, medium, high evidence strength). Conceptually, we modeled our initial tool in the same way; grouping criteria by domain and having a series of evidence levels. We conducted a total of 10 interviews. Informants reflected on different potential domains (e.g., elements of the research design) and listed specific ways that they felt research and evidence quality differed in implementation research from clinical trials. Among factors highlighted were a need to consider the context and specification of strategies, criteria specific to implementation outcomes, and consideration of the equity impact of implementation strategies on the health outcome under consideration.

Based on these results, we structured our initial tool along six domains: overall effectiveness, study design quality, implementation outcomes, equity impact, strategy specification, and a bundled strategies domain. Each domain included a set of criteria considered within each domain. For example, criteria for the implementation outcomes domain included operationalization of implementation outcomes; validity and reliability of measure used; significance and direction of effect for quantitative outcomes; and reported effects as beneficial, neutral, or harmful. We also developed and defined five evidence levels with associated recommendations: best practice strategy, promising strategy, emerging strategy, undetermined strategy, non-recommended strategy. As an example, promising strategies were described as demonstrating mostly positive outcomes that may need more rigorous examination to ensure they are having the intended effect or are generalizable to a wider context. Practitioners would be recommended to take caution when using a promising strategy in practice and ensure it is having a similar outcome as demonstrated in the original research.

### Modified Delphi

For the Delphi Round 1, we recruited from a pool of 68 experts. Two individuals responded stating their inability to participate, with one participant suggesting a replacement. Forty-one participants completed the survey, and two participants partially completed the survey for a total of 43 participants (63% response rate). For the Delphi Round 2, we recruited among the responders from Round 1 with no refusals to participate and no partial responses. Thirty participants in total completed the Round 2 survey (70% response rate). Respondent characteristics are provided in Table 1 for both Delphi Rounds. Briefly, one half of Respondents in both rounds self-identified as women (55.8%; 50% in rounds 1 and 2 respectively), with the majority white (83.7%; 80%) and not Hispanic or Latino (86%; 100%). Most respondents worked in academic settings (81.4%; 80%), with most working in HIV in round 1 but not round 2 (83.7%; 36.7% respectively). The highest number respondents had 11–20 years of experience in their area of expertise (44.2%; 43.3% respectively), and three quarters reported experience with leading implementation research projects (76.7%; 73.3%). Both complete and partially complete responses are included in the analyses.

#### Delphi Round 1.

Table 2 presents the quantitative outcomes regarding whether the participant believed that domains should be added, dropped, or combined. More than half (58%) of participants thought no new domains should be added, while 44% of participants thought domains should be dropped or combined. When examining the evidence levels, 79% of individuals felt that no additional evidence levels were needed, while 47% thought one or more of the evidence levels could be dropped or combined.

Table 3 summarizes open-ended responses with example quotes for domains and evidence levels that were commented on most often. When reviewing the qualitative responses of those who indicated a domain should be added, most respondents suggested adding specific criteria or wanted greater clarity in how the domains and criteria within domains were defined. For example, regarding the equity domain, individuals desired greater clarity, operationalization, and description of how equity is being considered and evaluated. Of these, four sought greater clarity of equity-related outcomes, and six recommended inclusion of equity metrics or different ways of operationalizing equity. Three participants felt equity should be examined in combination with implementation outcomes. Three suggested greater consideration of community partnership development and inclusion of the target population in the development of the strategy or design of a study. Finally, participants recommended combining promising, emerging, and/or undetermined as levels of evidence and better specifying and operationalizing the levels.

Briefly, we revised the structure of our tool along five domains: study design, implementation outcomes, study rigor and limitations, strategy specification, and equity impact. These domains each included a revised set of criteria. For example, based on the recommended additions to the study design and rigor domain, we split this domain into two domains: 1) study design; and 2) study limitations. We considered several of the comments on dropping equity but ultimately opted to keep this domain, relax the criteria, and heavily refine the description. Other cross-cutting changes included combining the criteria for bundled strategies and strategy specification. We combined two of the evidence levels (emerging and undetermined) and revised the definitions to include: best practice, promising practice, needs more evidence, and harmful.

#### Delphi Round 2.

For the second round of the Delphi, we asked respondents to confirm major changes to the tool based on the first round of the Delphi (Table 2), and have respondent evaluate our proposed process for applying the criteria. Most respondents agreed with changes to the domains and evidence levels although there remained some commentary on the equity domain. When examining the open-ended responses among those disagreeing with the changes to the equity domain, we grouped responses into individuals that did not agree with the domain (i.e., a hard no to the revisions) and others who still had additional suggestions for the domain but approved of the domain overall (i.e., a soft no with suggested revisions; Table 3). Based on these responses, we finalized the domains and made several additional adjustments to the definitions of equity including defining which target populations can be considered in determining whether the strategy has a positive equity impact or not. Finally, we revised our process for applying the rubric based on the recommendation to apply the criteria across each domain in addition to giving an overall rating. While this did increase time in the review process, this change allowed us to still provide information on how strategies rate across all domains, enabling researchers and practitioners to compare how strategies rate on different domains or select a strategy that is strong in a specific domain, like equity supporting for example.

### Pilot Application to PrEP Implementation Strategies

To ensure a consistent, high-quality process for applying criteria to research studies examining implementation strategies, we initially piloted the rubric with existing studies on implementation strategies to promote the uptake of evidence-based HIV services. At the conclusion, we were able to achieve 90% reliable application of the criteria, resulting in dropping some criteria and clarifying other criteria and their application. Two members of the ISCI team then applied the rubric to a set of 18 implementation strategies identified through an ongoing systematic review designed to promote uptake of PrEP in a second pilot application, achieving 98% reliability. Among the 18 strategy studies, summarized in Table 4, one was assigned an overall rating as Best Practice and the remaining were assigned as Needs More Evidence. The primary domains where strategies failed to exceed the Needs More Evidence criteria were in Research Design as well as Study Rigor and Limitations. This was largely because these studies only utilized post-implementation assessment, were intended as pilot or feasibility studies, or were conducted only at a single site. Given the early state of the implementation research related to PrEP implementation in the US, we felt that this mix of ratings was relatively appropriate. While the domains that have parallels in other rating systems resulted in relatively low ratings among our studies, we observed a good mix of ratings on domains unique to our tool and implementation research (i.e., strategy specification and equity) at the Best, Promising, and Needs More Evidence levels, suggesting these domains are sufficiently discerning among the existing set of studies.

A summary of major changes to the rubric and criteria are summarized in Table 5. The final domains and evidence-levels are provided in Table 6. The final rubric with domains, criteria, evidence levels, and application instructions are presented in Table 7.

## Discussion

To our knowledge, this is the first set of criteria to evaluate level of evidence for implementation strategies to establish evidence quality and serve as a basis for recommendations to practitioners. Our Best Practice rubric was initially informed by criteria used for interventions and interviews, refined by a Delphi, and then piloted with implementation strategies. This process yielded a rating scale (i.e., best, promising, needs more evidence, and harmful) and domains (e.g., study design, implementation outcomes, rigor, and limitations), which are common to other tools and rubrics. Yet, implementation research’s unique system-level focus required tailoring to our rubric. For instance, we have outlined criteria for the research design domain that considers the realities of where implementation research is conducted and does not require blinding or randomization for strategies to be considered the highest rating. To help define the criteria for these domains, we used Proctor’s recommendations for strategy specification and for implementation outcomes, as well as current commentary on research rigor in implementation science (1, 6, 22). While these helped provide structure and specific criteria at each of the evidence levels, in conducting the pilot we noted missing information which sometimes made it difficult to evaluate the research. We recommend using Standards for Reporting Implementation Studies (StaRI) guidelines as well as Proctor’s recommendations for strategy specification when reporting implementation research to help report the needed details to evaluate the research and for potential practitioners to understand what resources and efforts are needed for implementation strategies (1).

In addition to being a new resource for implementation science, to our knowledge this is also the first evidence rating criteria that considers the potential to improve equity in a health issue. Because implementation science directly impacts communities with the potential to improve or exacerbate inequities, HIV included, experts reiterated that equity was a critical domain to include. However, our work demonstrates a lack of consensus in the implementation science field about what equity in implementation science means. We have emphasized community engagement in the research process, a research focus on populations experiencing inequities, as well as equity in outcomes as a means to encourage attention to and improvement in HIV-related inequities as many in the field have advocated (23–25). We recognize that no single implementation strategy (or intervention) is going to adequately address the deeply rooted structural determinants, like racism and homophobia, which keeps inequities entrenched. However, it is our hope that by including equity improvement as criteria to be considered the highest quality research, we can bring additional attention to and encourage equity in HIV outcomes in the US.

Our rubric and criteria are designed to discern among studies specific to HIV implementation research, which is a rapidly growing field. There are other heath areas, such as cancer, for which there are more studies leveraging more rigorous research designs to evaluate implementation strategies (26, 27). We encourage others who may consider using this rubric in their area of implementation science to consider the specific criteria within each of the domains and at each of the evidence-levels to ensure that it appropriately discerns among available studies before application. Conversely, we received many suggestions about more stringent criteria that participants felt like should be included that we were not able to include because it would have resulted in few-to-no strategies identified as best practice. US focused HIV implementation science is still in its adolescence, with many pilots and full-fledged trials underway but not yet published. It is our hope that in the future, we will be able to include more stringent criteria within the rubric so that the needed evidence quality improves over time within HIV implementation research.

There are some notable limitations to the processes used to develop the Best Practice rubric and the criteria themselves. We used a Delphi modified approach to develop the rubric and criteria. Our use of this method did not result in consensus, but instead resulted in an approximation of consensus. In addition, we were not able to elicit the opinions about the appropriateness of the rubric and tool from the perspective of front-line implementers on balance with those of the research community. We hope to address this in future iterations of this work.

We envision several future directions for this tool with implications for both researchers and practitioners. Systematic reviews of HIV-related implementation strategies are currently underway (28). The next phase will entail applying these criteria to implementation strategies identified through these reviews and developing a compendium of strategies, with the intention of supporting strategy dissemination with best or promising evidence for their adoption and scale up. We recognize that a rating and recommendation is not sufficient to support uptake, and a complementary dissemination effort is underway to provide the needed information and materials for adoption and penetration. Our criteria and rating system will also yield benefits for researchers conducting HIV implementation research. Along with the systematic review, it will identify strategies for which there is already good evidence, as well as strategies that hold promise but would benefit from additional research and additional evidence supporting their effectiveness. Researchers can also use these criteria in designing studies of new strategies so that they can score better on these criteria.

## Conclusion

For practitioners to fully benefit from research developing and testing implementation strategies targeting HIV services, clear evaluation criteria and recommendations are needed to assess which strategies are the most likely to have benefit and impact. Our process for developing a rubric and criteria yielding domains and criteria specific and appropriate for implementation research that can be used to evaluate evidence quality in HIV-related implementation strategies. This rubric includes recommendations for practitioners about strategies for which there is best evidence and recommendations for research about strategies for which more evidence is needed. Establishing criteria to evaluate implementation strategies advances implementation science by filling a much-needed gap in HIV implementation research which can be extended to other areas of implementation science.

## Figures and Tables

**Table 1.** Participant Characteristics

	Round 1		Round 2	
	N	(%)	N	(%)
I work primarily in… (Select all that apply)				
Clinical settings	15	(34.9)	2	(6.7)
Public health settings	12	(27.9)	3	(10.0)
Academic settings	35	(81.4)	24	(80.0)
Federal government	5	(116)	3	(10.0)
Other	1	(2.3)	2	(6.7)
I spend a significant percent of my time (select all that apply)				
Providing services directly to patients or clients	10	(23.3)	6	(20.0)
Researching interventions, practices, or policies for scientific purposes	39	(90.7)	24	(80.0)
Evaluating interventions, practices, or policies for organizational quality improvement	23	(53.3)	10	(33.3)
Determining and providing funding for health services	7	(16.3)	2	(6.7)
Developing or implementing policies that influence the provision of health services	6	(14.0)	4	(13.3)
Other	1	(2.3)	0	(0.0)
I work in the following areas… (Select all that apply)				
HIV	36	(83.7)	11	(36.7)
Mental Health	18	(41.9)	10	(33.3)
Primary Care	22	(51.2)	10	(33.3)
Substance Misuse	10	(23.3)	6	(20.0)
Women’s Health	14	(32.6)	8	(26.7)
Social Services	7	(16.3)	1	(3.3)
Another Area of Health	11	(25.6)	9	(30.0)
How would you describe your level of knowledge of implementation science?				
I know almost nothing or only the basic components of implementation science	2	(4.7)	1	(3.3)
I know a moderate amount about implementation science	12	(27.9)	5	(16.7)
I know a lot about implementation science	29	(67.4)	24	(80.0)
What would you say is your level of experience in implementation research? (Select all that apply)				
I have not formally been involved in implementation research	0	(0.0)	0	(0.0)
I have supported implementation research projects	25	(58.1)	10	(33.3)
I have led implementation research projects	33	(76.7)	22	(73.3)
I have been a partner in implementation research	18	(41.9)	11	(36.7)
How many years of experience do you have in your field?				
0–10 years	5	(116)	4	(13.3)
11–20 years	19	(44.2)	13	(43.3)
21–30 years	12	(27.9)	5	(16.7)
31 years of more	7	(16.3)	8	(26.7)
What is your race? (Select all that apply)				
Asian	5	(116)	6	(20.0)
Black or African American	2	(4.7)	0	(0.0)
White	36	(83.7)	24	(80.0)
American Indian, Alaska Native, Native Hawaiian, Pacific Islander, or Other	2	(4.7)	3	(10.0)
Prefer not to respond	1	(2.3)	1	(3.3)
What is your ethnicity?				
Hispanic or Latino	1	(2.3)	0	(0.0)
Not Hispanic or Latino	37	(86.0)	30	(100)
No response	5	(116)	0	(0.0)
How do you describe yourself? (Select all that apply)				
A man	17	(39.5)	13	(43.3)
A woman	24	(55.8)	15	(50.0)
Nonbinary	1	(2.3)	1	(3.3)
Prefer not to respond	1	(2.3)	1	(3.3)
Total	43	(100)	30	(100)

**Table 2.** Confirming Domain and Evidence Level Changes

**Delphi Round 1 Results**						
Should domains be…	Yes		No		Missing	
	N	(%)	N	(%)	N	(%)
Added?	17	(39)	25	(58)	1	(2)
Dropped?	6	(14)	35	(81)	2	(5)
Combined?	13	(30)	29	(67)	1	(2)
Should evidence levels be.	Yes		No		Missing	
	N	(%)	N	(%)	N	(%)
Added?	5	(12)	34	(79)	4	(9)
Dropped?	8	(19)	31	(72)	4	(9)
Combined?	12	(28)	29	(67)	2	(5)
**Delphi Round 2 Results**						
Do you agree with the revisions?	Yes		No		Missing	
	N	(%)	N	(%)	N	(%)
Study Design and Limitations	25	(85)	5	(15)	0	(0)
Dropped Bundled Strategies	26	(87)	4	(13)	0	(0)
Equity Impact	23	(75)	7	(25)	0	(0)
Combined Evidence Levels	27	(90)	3	(10)	0	(0)

**Table 3.** Delphi Open-ended Responses

**Round 1 Suggestions**	
Theme	Quote
Combine Equity with other Domains	Not to drop equity - it should be somewhere -, but equity and reach (from implementation outcomes have considerable overlap). This could be a single implementation outcome domain.
Combine Bundled Strategies with Other Domains	“Nearly all implementation interventions are complex health interventions and hence bundled or multi-component. Any considerations related to bundled strategies should be generalized and applied to all strategies …”
Clarify Overall Evidence of Effectiveness	Overall evidence of effectiveness is generally understood as average effect size estimates, which are largely meaningless and unhelpful when studying complex health interventions.. strong, high-quality designs for complex health interventions require application of the core function/form framework, allowances for adaptation and tailoring and assessment of fidelity to function (not form).. **Research answering the simple (simplistic) question “is intervention X effective” offers little value in implementation science, insofar as the dominant answer is “sometimes” or “it depends**.”
Merge Medium Levels (promising/emerging)	“I struggle a little with **the distinction between Promising Strategies and Emerging Strategies**. I suspect the distinction might be between the number of studies? but the conclusion seems to be the same regardless.”
**Round 2 Open-ended Responses on Equity Domain Changes**	
Hard no on equity	I still don’t see why this isn’t considered as an implementation outcome. It seems like a normative decision to separate the domain.This just seems like an unnecessary complexity.
Soft no on equity with suggested changes	I agree with keeping it separate but think there are two separate domains: (1) the actual reach to specific populations. This can be covered elsewhere and (2) the intentionality of the strategy to reach a specific marginalized population (including involving marginalized individuals/population representatives in strategy development and research design). I think this is very reasonable to have as a separate domain. I would give examples of target populations, but not define them or leave it to the CDC to define. Some of the most marginalized populations may be identified through community engaged research and implementation science.
	Might consider using a more well-established definition of health equity research-for example, something from CDC or NIH. Also seems like the partner engagement would be more under engagement (broadly speaker) not explicitly called out under health equity..

**Table 4.** Rubric and criteria application to implementation science studies focused on PrEP

First Author	Year	Article Title(s)	Study Design	Outcomes	Rigor	Specification	Equity	Overall
Brant	2020	Integrating HIV PreExposure Prophylaxis into Family Planning Care: A RE-AIM Framework Evaluation (29)	Needs More	Best	Needs More	Best	Promising	Needs More
Buchbinder	2019	Getting to Zero San Francisco: A Collective Impact Approach (30)	Needs More	Needs More	Needs More	Needs More	Best	Needs More
Bunting	2019	A Guide for Designing Student-Led, Interprofessional Community Education Initiatives About HIV Risk and Pre-Exposure Prophylaxis (31)	Best	Needs More	Needs More	Best	Promising	Needs More
	2020	Using a student-led, community-specific training module to increase PrEP uptake amongst at-risk populations: results from an exploratory pilot implementation (32)						
Burns	2021	Meet Me Where I Am: An Evaluation of an HIV Patient Navigation Intervention to Increase Uptake of PrEP Among Black Men Who Have Sex with Men in the Deep South (33)	Needs More	Best	Needs More	Best	Needs More	Needs More
Chen	2014	Clinical effectiveness and cost-effectiveness of HIV pre-exposure prophylaxis in men who have sex with men: risk calculators for real-world decisionmaking (34)	Needs More	Needs More	Promising	Needs More	Promising	Needs More
Clement	2019	Advancing the HIV Pre-Exposure Prophylaxis Continuum: A Collaboration Between a Public Health Department and a Federally Qualified Health Center in the Southern United States (35)	Needs More	Best	Best	Best	Best	Needs More
Coleman	2020	Integrated Pharmacy and PrEP Navigation Services to Support PrEP Uptake: A Quality Improvement Project (36)	Best	Best	Best	Best	Best	Best
Gregg	2020	Implementation of HIV Preexposure Prophylaxis in a Homeless Primary Care Setting at the Veterans Affairs (37)	Needs More	Best	Needs More	Best	Best	Needs More
Havens	2019	Acceptability and feasibility of a pharmacist-led HIV pre-exposure prophylaxis (PrEP) program in the Midwestern United States (38)	Needs More	Best	Needs More	Promising	Promising	Needs More
Horack	2020	Pre-Exposure Prophylaxis in a Reproductive Health Setting: A Quality Improvement Project (39)	Needs More	Needs More	Needs More	Needs More	Needs More	Needs More
Hoth	2019	Iowa TelePrEP: A Public-Health-Partnered Telehealth Model for HIV PreExposure Prophylaxis (PrEP) Delivery in a Rural State (40)	Needs More	Best	Needs More	Best	Promising	Needs More
Khosropour	2020	A Pharmacist-Led, Same-Day, HIV PreExposure Prophylaxis Initiation Program to Increase PrEP Uptake and Decrease Time to PrEP Initiation (41)	Needs More	Best	Needs More	Promising	Promising	Needs More
Lopez	2020	Implementation of pre-exposure prophylaxis at a community pharmacy through a collaborative practice agreement with San Francisco Department of Public Health (42)	Needs More	Best	Needs More	Best	Best	Needs More
Pathela	2020	The HIV Pre-exposure Prophylaxis (PrEP) Cascade at NYC Sexula Health Clinics: Navigation is the Key to Uptake (43)	Needs More	Best	Needs More	Needs More	Promising	Needs More
Roth	2020	Integrating HIV Preexposure Prophylaxis With Community-Based Syringe Services for Women Who Inject Druges: Results from the Project SHE Demonstration Study (44)	Needs More	Best	Needs More	Best	Promising	Needs More
Saberi	2018	A Simple Pre-Exposure Prophylaxis (PrEP) Optimization Intervention for Health Care Providers Prescribing PrEP: Pilot Study (45)	Needs More	Best	Needs More	Best	Needs More	Needs More
Tung	2018	Implementation of a community pharmacy-based pre-exposure prophylaxis service: a novel model for pre-exposure prophylaxis care (46)	Needs More	Best	Needs More	Best	Needs More	Needs More
Wood	2018	Project ECHO: telementoring to educate and support prescribing of HIV pre-exposure prophylaxis by community medical providers (47)	Best	Needs More	Needs More	Promising	Needs More	Needs More

**Table 5.** Summary of rubric and criteria revisions after each development phase

Initial review and Interviews	Delphi Round 1	Delphi Round 2	Piloting
Initial proposed domains: overall effectiveness, study design quality, implementation outcomes, equity impact, strategy specification, & bundled strategies Initial proposed evidence levels: best practice, promising, emerging, undetermined, & non-recommended strategy Initial proposed criteria for each domain	Dropped overall effectiveness domain Split study design quality domain into study design and rigor and limitations domain Revised equity domain description Combined emerging and undetermined evidence levels and renamed as more evidence needed Reduced criteria across all domains at all evidence-levels	Confirmed domains Revised equity domain descriptions Confirmed evidence levels Reduced criteria across all domains at all evidence-levels Revised application process	Revised application process Revised criteria definitions

**Table 6.** Descriptions of Criteria Domains and Evidence Levels

Criteria Domains	
Domains	Descriptions
Study Design	The elements of study design(s) used to evaluate a strategy, primarily the use of a comparison group and assessment before (pre) and after (post) a strategy is used.
Implementation Outcomes	The effect and effect direction of deliberate and purposive actions to implement new treatments, practices, and services.
Rigor & Limitations	Aspects of the study design that may limit the validity or generalizability of the results.
Strategy Specification	The level of specificity about the strategy for purposes of reproducibility.
Equity Impact	The impact of the strategy among target populations disproportionately impacted by the HIV epidemic.
Evidence Levels	
Evidence Levels	Descriptions
Non-Recommended	Strategies demonstrate a harmful effect. These would not be recommended for practice or scientific investigation.
Needs More Evidence	Strategies demonstrate null results or positive results from preliminary research. These strategies would be recommended for additional research with more rigorous research designs.
Promising	Strategies demonstrate positive outcomes but may be limited on some aspects of criteria domains. These strategies would be recommended to practitioners but may require careful monitoring to ensure similar outcomes.
Best	Strategies demonstrate positive outcomes from rigorous research. These strategies would be recommended to practitioners for the barriers they are intended to address.

**Table 7.** Evidence Levels and Criteria of Evidence Levels for each Domain

Criteria Domains	Evidence Levels	Criteria for Evidence Levels
Instructions for application: The criteria within the Best Practice evidence level must be met to be assigned that evidence level otherwise it should be assigned as More Evidence Needed. All studies should be rated as either Best Practice or More Evidence Needed.		
Study Design	Best Practice	Pre/post assessment of implementation outcomes OR has a comparison group Example Designs: Multi-site Hybrid II OR III trials, natural experiments, within-site repeated measures designs
	Promising	
	More Evidence Needed	Identifies as a feasibility or pilot study Post evaluation only with no comparison group Simulation Studies Example Designs: Pilot studies, post assessment of implementation of strategy only, hybrid type I studies
	Non-Recommended	
Instructions for application: Results related to implementation outcomes from quantitative or qualitative studies can be considered. If any outcome is harmful, it should be assigned as Non-recommended, and the reviewer can stop. Consider the primary implementation outcome from the study targeted by the implementation strategy - this should be at the clinician level. If no primary outcome is identified in the study, consider the most proximal implementation outcome to the strategy. Then consider the criteria for the Best Practice evidence level. If all criteria are met, the reviewer can assign that evidence level and stop. Otherwise, consider which criteria are met at the More Evidence Needed and assign at that level. If any outcomes is harmful, it should be assigned as Non-recommended.		
Implementation Outcomes and Effects	Best Practice	Primary implementation outcome is operationalized - meaning a definition of how the outcome variable is defined, measured, or calculated Effect from primary outcome is reported as positive or beneficial. Studies with post only measurement, if the outcome is accomplished, then the outcome can be considered positive Quant studies with “Best Practice” design that is either predominantly quantitative or mixed-method: Has a significant positive effect on at least one targeted implementation outcome (p<.05).
	Promising	
	More Evidence Needed	Effect from primary outcome is reported as null or not achieved as intended Quant studies with “Best Practice” design that is either predominantly quantitative or mixed-method: Has a significant positive effect on at least one targeted implementation outcome, but at less stringent criteria (p<.1). Implementation outcome(s) (e.g., adoption, feasibility) are mentioned but perhaps not operationalized
	Non-Recommended	Demonstrates harmful implementation outcomes - primary or secondary
Instructions for application: Consider the additional design criteria of the study to evaluate for major limitations to validity or generalizability of the research. For qualitative studies, criteria only need be described (no determination of adequacy or quality necessary). For mixed method studies with a primary method (i.e., primarily quantitative or primarily qualitative), criteria need only be met for the primary method. For mixed method studies with balanced methods (i.e., quantitative and qualitative methods are equal), criteria for both methods must be met. In addition, methods in mixed method studies must complement each other. For the appropriate study design begin at the Best Practice evidence level. If all appropriate criteria are met at that level, the reviewer can stop and assign the rating. Otherwise consider the criteria for the Promising evidence level. If the study meets all the criteria at the Promising evidence level, then the reviewer can stop and assign the rating. Otherwise assign the level Needs More Evidence.		
Study Rigor and Limitations	Best Practice	Quant studies: Approaches to bias minimization used (e.g., randomization, matching, statistical comparison of intervention and control sampling, sample weighting, etc.); Study includes multiple sites or replication has occurred Qual studies: Description of data collection procedures, management, and transcription; Description of analysis, analytic codes, and code definitions; Intercoder reliability or member checking conducted during analyses if multiple coders are used Mixed Method Studies: Methods complement one another
	Promising	Quant studies: Approaches to bias minimization used (e.g., randomization, matching, statistical comparison of intervention and control sampling, sample weighting, etc.) Qual studies: Description of data collection procedures, management, and transcription; Description of analysis, analytic codes, and code definitions; Intercoder reliability or member checking conducted during analyses if multiple coders are used Mixed Method Studies: Methods complement one another
	More Evidence Needed	Quant studies: Approaches to bias minimization not used (e.g., randomization, statistical comparison of intervention and control samples, sample weighting, etc.); Study conducted at a single site with no replication Qual studies: Inadequate description of multiple aspects of the research design including data collection approach and analysis Mixed Method Studies: Unclear whether methods complement one another
	Non-Recommended	
Instructions for application: Evaluate quality of strategy specification, context description, and connection to identified determinants. The reviewer may infer elements of strategy specification if they are not described using these terms overtly. For the appropriate strategy design begin at the Best Practice evidence level. If all appropriate criteria are met at that level, the reviewer can stop and assign the rating. Otherwise consider the criteria for the Promising evidence level. If the study meets all the criteria at the Promising evidence level, then the reviewer can stop and assign the rating. Otherwise assign the evidence level Needs More Evidence.		
For strategy specification Proctor’s recommendations for implementation strategy operationalization should be used defined as:		
Actor – Identify who enacts the strategy. Who is responsible for enacting the strategy? (3 or 2 on our systematic review scale)		
Action – The specification actions, steps, or processes that need to be enacted. In a general sense, what does the strategy entail? While it’s not expected that the authors provide every detail, the reader should be able to consider whether the strategy could potentially be used in their context. Ideally, they would do more reading to make a final determination and get more details.		
Target – Unit of analysis for measuring implementation outcomes. What is expected to change because of the strategy? All included strategies in this review should meet this criteria.		
Temporality – Timing or sequencing of strategy. When should the strategy be used? For this element, the reviewer can assume that the strategy would naturally be employed immediately or any time if appropriate. However, some strategies may require specific timing, e.g., getting collaborative agreements in place before implementing a referral program. In these cases, it should be described in the paper.		
Dose - Dose or intensity of strategy including frequency or length of time. How often should the strategy be used? Similar to above, the reviewer can assume the strategy should naturally be used all the time or always if appropriate. However, some strategies may involve a specific dose, e.g., educational programs. In these cases, it should be described in the paper.		
Strategy Specification	Best Practice	
		Context: Studies identify the context(s) in which the strategy was implemented including type(s) of agencies and demographics of participants at the deliverer and recipient-levels Barriers/Facilitators: Studies discuss the potential barriers and facilitators in which implementation has been trialed (most likely located in the intro) Strategy Selection Rationale: There is a rationale for how the strategy works and why it should be effective (i.e., theoretical or empirical connection to barrier(s)) Strategy Elements: **All** strategy elements are mentioned or can be inferred as appropriate. For blended or multi-component strategies: Justification for the blending of strategies or utilization
	Promising	Context: Studies detail the context(s) in which the strategy was implemented including type(s) of agencies and demographics of participants at the deliverer and recipient-levels Barriers/Facilitators: Studies discuss the potential barriers and facilitators in which implementation has been trialed Strategy Elements: Majority (3 of 5) of strategy elements mentioned or can be inferred as appropriate, but some aspects may be insufficiently specified Unclear rationale for how the strategy works or why it should be effective For blended or multi-component strategies: Lacks justification for the blending of strategies or utilization
	More Evidence Needed	Context: Studies do not detail the context(s) in which the strategy was implemented including type(s) of agencies, demographics of participants at the deliverer and recipient-levels Barriers/Facilitators: Studies do not discuss potential barriers and facilitators to implementation Unclear rationale for how the strategy works or why it should be effective Majority of strategy elements are not specified For blended or multi-component strategies: lacks justification for the blending of strategies or utilization
	Non-Recommended	
Instructions for application: Examine the equity impact of the strategy based on the communities targeted in the research, the outcomes for these groups at either the level of implementation or health outcomes, and their engagement in the research process. As a reminder, target populations for HIV prevention and treatment include but are not limited to men who have sex with men (e.g., gay and bisexual men), African Americans, Latinx individuals, people who inject drugs, or transgender individuals. Other populations may be considered if an inequity has been identified.		
First check to make sure there are no negative effects that would increase inequities. If yes, then assign as non-recommended and stop. Then begin at the Best Practice evidence level for the appropriate strategy design. If all appropriate criteria are met at that level, the reviewer can stop and assign the rating. Otherwise consider the criteria for the Promising evidence level. If the study meets all the criteria at the Promising evidence level, then the reviewer can stop and assign the rating. Otherwise assign the evidence level Needs More Evidence.		
Equity Impact	Best Practice	Includes target populations experiencing inequities Has a positive effect on target populations that improves equity or reduces disparities specifically on a target population Frames research in terms of equity promotion (e.g., describes inequity in introduction or proposes aims to reduce inequities or is informed by an equity framework) Uses formative research with community, directly engages communities during the study, OR strategy improves interaction with community/patients.
	Promising	Includes target populations experiencing inequities Examines differences in outcomes based on target populations Has positive outcomes overall but does not necessarily improve equity
	More Evidence Needed	Does not focus on health equity Excludes populations experiencing inequity without rationale
	Non-Recommended	Found to have a negative effect on health equity (i.e., increases disparities)

## Data Availability

The Delphi dataset generated during the current study available from the corresponding author on reasonable request.
